# Conceptualization of molecular findings by mining gene annotations

**DOI:** 10.1186/1753-6561-7-S7-S2

**Published:** 2013-12-20

**Authors:** Vicky Chen, Xinghua Lu

**Affiliations:** 1 Department of Biomedical Informatics, University of Pittsburgh, 5607 Baum Blvd, Pittsburgh, PA 15206, USA

## Abstract

**Background:**

The Gene Ontology (GO) is an ontology representing molecular biology concepts related to genes and their products. Current annotations from the GO Consortium tend to be highly specific, and contemporary genome-scale studies often return a long list of genes of potential interest, such as genes in a cancer tumor that are differentially expressed than those found in normal tissue. It is therefore a challenging task to reveal, at a conceptual level, the major functional themes in which genes are involved. Presently, there is a need for tools capable of revealing such themes through mining and representing semantic information in an objective and quantitative manner.

**Methods:**

In this study, we utilized the hierarchical organization of the GO to derive a more abstract representation of the major biological processes of a list of genes based on their annotations. We cast the task as follows: given a list of genes, identify non-disjoint, functionally coherent subsets, such that the functions of the genes in a subset are summarized by an informative GO term that accurately captures the semantic information of the original annotations.

**Results:**

We evaluated different metrics for assessing information loss when merging GO terms, and different statistical schemes to assess the functional coherence of a set of genes. We found that the best discriminative power was achieved by using a combination of the information-content-based measure as the information-loss metric, and the graph-based statistics derived from a Steiner tree connecting genes in an augmented GO graph.

**Conclusions:**

Our methods provide an objective and quantitative approach to capturing the major directions of gene functions in a context-specific fashion.

## Background

Contemporary "omics"-scaled studies often produce a large volume of data, where the amount of information overwhelms human comprehension and defies manual inspection. For example, the Cancer Genome Atlas (TCGA) [1] project characterizes thousands of tumor samples in terms of gene expression, genetic variations, and other molecular biology aspects. Since a cancer always results from perturbations of multiple biological processes [2], a whole list of differentially expressed genes in a tumor inevitably reflects a mixture of genes responding to distinct signals. It becomes an increasingly important task to de-convolute the signals reflected by molecular data and to represent information at a conceptual level by answering questions such as the following: "Given a list of differentially expressed genes from a tumor, which major biological processes are perturbed in the tumor?" In this study, we aim to develop information-theory-based and quantitative approaches to reveal the *major *functional themes among a gene list in a case-specific manner, and to summarize the findings using *informative *concepts.

We formulate the task as follows: divide the genes in a list into non-disjoint subsets, such that genes in each subset participate in coherently related biological processes, and such that the overall functional theme of a subset can be represented with an informative concept in a bio-ontology. The goal of finding *major *and *informative *biological processes requires one to strike a balance such that a subset should include as many genes as possible in order to reflect a major theme of the genes list, and yet the concept representing the functional theme of the subset should be as specific as possible. Although there is a large body of literature on using bio-ontologies to represent the information derived from genome-scale experiments [3-7], few studies specifically address the task of identifying *informative *concepts to represent the information from experimental data in a case-specific, objective, and quantitative manner.

Generally speaking, a method that addresses the above task should have the following capabilities: 1) dynamically selecting concepts that are best suited to represent the functional themes specific for a given gene list and 2) assessing the suitability of a concept to represent the information of the genes within a subset as specifically as possible. One can utilize the hierarchical organization of well-formed bio-ontologies to achieve the first capability. The second requirement is more challenging, in that it requires a quantitative measure to assess the specificity of the concept and assess the statistical significance as an objective criterion to guide the search for the balance point between capturing the major theme of genes while maintaining the specificity of the theme.

The Gene Ontology (GO) [8], a controlled vocabulary consisting of molecular biology terms (concepts) related to genes, is the most widely used bio-ontology for representing the information derived from genome-scale experiments, particularly about aspects of the biological process (BP). Currently, a common approach to finding a functional theme from a gene list is to assess whether any GO terms are enriched among the annotations associated with a gene list [9-12]. However, annotations by the GO Consortium are usually highly specific, and it is not uncommon to find a set of specific GO terms enriched in a gene list where each term covers only a small number of genes, thus failing to reveal the *major *biological process. Aware of the need for more general concepts, the GO Consortium provides a set of general GO terms, referred to as GO slim [8], that represent high-level biological concepts. There are also software tools that map/associate genes to concepts in the GO slim subsets [13-15]. As will be shown, these terms tend to be too general; more importantly, this small set of predefined GO terms may not meet the need of revealing functional themes in a case-specific fashion with balanced generality and specificity. Besides the GO enrichment analysis, another widely used approach for finding functional themes of a gene list is to assess whether members of certain predefined pathways, or gene signatures from databases such as KEGG [16] pathways and MSigDB [17], are enriched. However, such representations lack the ontological structure to support further reasoning by computing agents, and thus are unable to dynamically find the concepts best suited to represent the information of a gene list in a case-specific manner.

**Figure 1:**
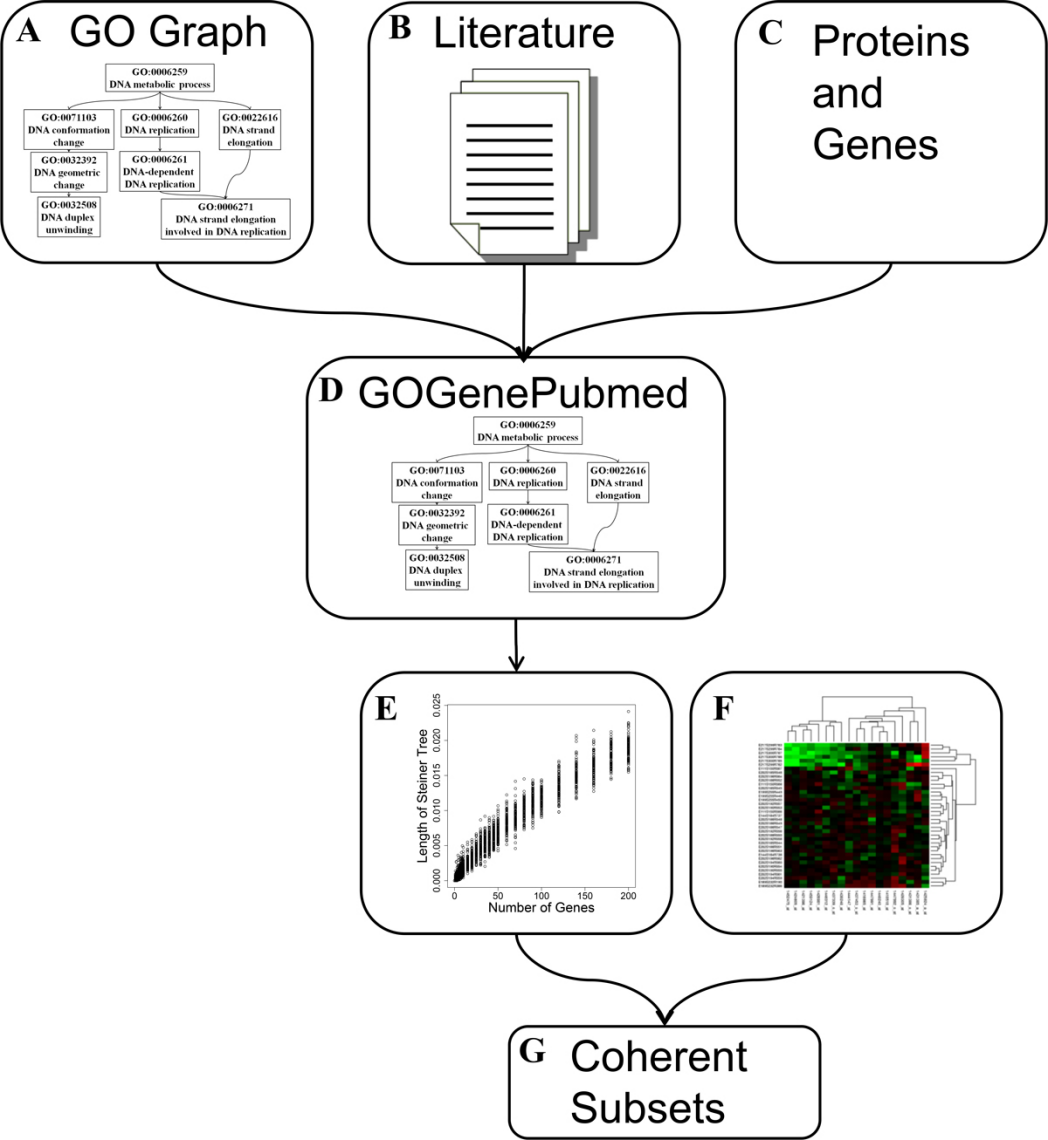
**Conceptual Overview of Research**. **A-C.** The ontological structure of the GO, protein annotations, and biomedical literature associated with genes were collected. **D.** The above information was combined to create an integrated graph (GOGenePubmed) that reflects the relationship among genes, their annotations, and the semantic relationships between GO terms. **E.** Based on this graph, statistical schemes were designed and simulation experiments were performed to establish statistical models for assessing the functional coherence of gene sets. **F-G.** When provided with a gene list from experiment **(F)**, the program can be used to search for coherent subsets among the list **(G)**.

In this study, we investigated a framework that utilizes the structure and semantic information of the GO to reveal major functional themes in a dynamic and gene-set-specific manner. We systematically studied different information-theory-based metrics to assess information loss when searching for suitable representations to summarize functional themes of gene sets. We further evaluated different statistical schemes to assess the functional coherence of a gene set summarized by a GO term. The conceptual overview of our research is shown in Figure 1.

## Methods

### Gene Ontology representation

We developed an object-oriented Python package that can store and represent the following information: 1) the structure of the GO; 2) the association of genes to the GO terms; and 3) the quantitative measure of information loss when the information represented by a specific concept (a GO term) is replaced by a more general ancestor concept. We modified and expanded a Python package, referred to as GOGrapher [18], previously developed by our group. We developed four hierarchically organized classes to represent the graph structure of the GO. Each type of graph contains a particular combination of information related to GO terms and utilizes a different measure to quantitatively reflect the information difference between a pair of GO terms. The GOGraph simply represents the GO terms and the IS_A relationships among the GO terms according to the definition of the Gene Ontology. The GOGeneGraph further stores information on associated gene products. The GOPubmedGraph stores information on associated PubMed records that can be used to construct the semantic context in which the concept represented by a term is discussed in biomedical literature. Finally, the GOGenePubmedGraph stores both types of information. GOGrapher package is publically available at the following URL: http://code.google.com/p/gographer/.

Data files containing the complete GO can be directly downloaded from the GO Consortium http://www.geneontology.org. The data used to generate our graphs was version 1.1.1961, downloaded on June 5, 2011. (For the ease of presentation, we generally refer to one of the above types of graphs as a "GO graph," and a reader can easily find out which type we are referring to based on the context of presentation.)

### Measuring information loss

GO nodes in the aforementioned graphs can be associated with genes and/or biomedical literatures; thus, each term contains information with respect to its genes and semantic context. When the information represented by a specific GO term is replaced by that of a more general term, information-theory-based measures can be used to assess how much information is lost. The graph representation of the GO conveniently allows us to represent such a loss as the weight of an edge between a parent-child pair of GO terms. In this study, we investigated two different measures as edge weights: 1) an information bottleneck (IB) -based measure, which assesses the loss of information with respect to the semantic context of the literatures associated with GO terms; and 2) a measure based on the information content (IC) of GO terms with respect to genes, which assesses the loss of information related to genes. The calculation of edge weights was based on the annotation data obtained from the UniProt multispecies annotation file downloaded on May 10, 2011, from the GO Consortium.

### Information bottleneck

To represent the semantic context when the concept of a GO term is discussed in literatures, we collected the titles and abstracts of PubMed records associated with each GO term. The PubMed records were preprocessed as described in our previous work [19], including by tokenization, stemming, and removing stop words and punctuations. Based on the IS_A relationship specified in the GO hierarchy, PubMed records are recursively propagated to ancestor nodes. To represent the semantic context of a GO term, we constructed a word-frequency-count vector, collecting tokens from the name and description of the GO term, and from the titles and abstracts of PubMed articles, to reflect the profile of words used to discuss the concept.

An IB-based metric can measure the amount of information loss when text documents are compressed into clusters [20,21]. In our previous work, we extended the metric to reflect the information loss when the semantic context of a GO term, represented as a word-usage profile, is represented by that of its parent node [19]. The information loss measured between a term and its parent is:

(1) $$\delta I\left(t_{i}\right)=p\left(t_{i}\right)D_{JS}\left(p\left(\vec{w}|t_{i}\right),p\left(\vec{w}|t_{p}\right)\right)$$

where

(2) $$p\left(t_{i}\right)=\frac{\left|t_{i}\right|}{\left|t_{root}\right|},$$

(3) $$D_{JS}\left(p\left(\vec{w}|t_{i}\right),p\left(\vec{w}|t_{p}\right)\right)=\pi_{i}D_{KL}\left(p\left(\vec{w}|t_{i}\right)||p\left(\vec{w}|t_{p}\right)\right),$$

(4) $$\pi_{i}=\frac{\left|t_{i}\right|}{\left|t_{p}\right|},$$

(5) $$D_{KL}\left(p\left(\vec{w}|t_{i}\right)||p\left(\vec{w}|t_{j}\right)\right)=\overset{\left|V\right|}{\underset{v=1}{\sum }}p\left(w_{v}|t_{i}\right)\log\frac{p\left(w_{v}|t_{i}\right)}{p\left(w_{v}|t_{j}\right)}.$$

In the equations, *D_JS _*represents the Jensen-Shannon divergence, |*t*| is the number of descendant terms of term *t*, *D_KL _*represents the Kullback-Leibler divergence, $p\left(\vec{w}|t\right)$ is the distribution of words associated with the GO term *t*, and *V *is the word vector. We used these formulae to calculate the information loss for each child term in relation to its parent.

### Information content

When GO terms are used to annotate genes, they contain the information with respect to genes. The amount of information a term has with respect to genes is commonly determined with an information-theory-based quantity referred to as information content (IC) [22-26]. The equation used to calculate the IC of a GO term is

(6) $$IC\left(t\right)=-ln\left(P\left(t\right)\right),$$

where *P*(*t*) is the number of gene annotation instances for the term *t *divided by the total number of gene annotation instances in the entire GO annotation database. Then, the difference in IC between a pair of parent-child nodes can reflect the amount of information that is lost with respect to genes when the child concept is collapsed into the parent node, and is calculated with:

(7) $$\text{dist}\left(t_{p},t_{c}\right)=\left|\text{IC}\left(t_{p}\right)-\text{IC}\left(t_{c}\right)\right|.$$

### Identify major functional themes from a gene list

When given a gene list, our goal is to separate the genes into non-disjoint subsets represented by informative GO terms that reflect the major functional themes. To this end, we developed the following procedure. 1) We represent the GO structure using a GO graph, and associate the genes to the GO terms according to their annotation file. 2) Terms without directly associated genes or any descendant with associated genes are then removed. 3) We begin merging leaf terms into their parent, starting with the terms that would result in the least information lost. If a term has more than one parent, it is merged into the parent that would most greatly minimize the total information lost. 4) After each merging, the parent term becomes a summarizing GO term annotating a set of genes, including the genes directly associated with the term, as well as the genes associated with its descendants. In other words, a summarizing GO term is annotating the genes from the subgraph beneath it. A merge will result in the loss of information with respect to genes as well as the loss of information with respect to the semantic context of the GO terms. 5) Applying the statistical analysis (to be discussed in the following subsections), we determine the information loss resulting from each merging and stop the merging of a GO term to its parent if it would lead to a set of genes that is deemed functionally incoherent. 6) Repeat steps 1 through 5. Eventually, this procedure will lead to a set of GO terms annotating the genes propagated from its subgraph. Thus, we achieve the goal of grouping genes into non-disjoint subsets, and the overall functional theme of each subset is represented by the final associated term.

### Statistical scheme for assessing the functional coherence of a subset

Given a set of genes produced during the merging process discussed in the previous subsection, we assess whether the functions of the genes are coherently related. This is determined by measuring how closely the genes are related to each other within a GO graph based on their function annotations: the more closely they are related to each other, the smaller the information lost during the merging process--and the greater the coherence of the gene set. We devised two graph-based statistics schemes to reflect the information loss created by the merging of the genes: 1) the total weight of the Steiner tree connecting the genes within the subgraph beneath a summarizing GO term; and 2) the total weight of an augmented Steiner tree connecting the genes within the subgraph beneath the summarizing term (see below).

Given a set of merged genes, we calculated its graph-based statistics using IC-based or IB-based information loss (see Section 2.2) as the edge weight. In the first statistics scheme, we found a Steiner tree [26,27] that connected the original GO terms annotating the genes within the subgraph and used the total weight of the edges as the coherence statistic. In the second statistical scheme, we introduced an edge between a pair of GO terms that annotate a common set of genes. This approach addresses the phenomenon that a gene can be annotated with distinct and yet closely related GO terms by different curators due to annotation inconsistency, which would artificially inflate the information loss reflected by the first statistic. Through adding an augmented edge, we aimed to collapse redundant annotations by assigning a small weight between them:

(8) $$d_{i,j}=\frac{d_{p_{5}}\in \left\{d_{G}\right\}}{\left|g_{i,j}\right|},$$

where *i *and *j *indicate two GO terms, ***d***_*G *_is the set of edge weights for all the edges in the GOGraph *G*, *d_P5_* is the value of the weight of the 5th percentile, and |***g***_*i, j*_| is the number of genes that are shared between the GO terms *i *and *j *[26]. After augmenting the graph, we then found a Steiner tree and used its total weight to reflect the total information loss.

### Statistical models for assessing coherence

Given a graph-based statistic reflecting overall information loss resulting from merging a set of genes under a GO term, we further devised statistical models to assess the functional coherence of the gene set. Here, we define a set of genes to be functionally coherent if the information loss is significantly less than that derived by merging a similarly sized set of randomly selected genes. To this end, we performed a series of simulation experiments drawing random gene sets, and developed statistical models (which can also be used to assess the significance of the graph-based statistics for each gene) to represent the distributions of random gene sets.

Since the graph-based statistics for a subset of genes can vary according to the size of the subset, we randomly drew gene sets ranging from 5 to 200 genes, with varying step sizes. To establish a distribution of statistics for random sets of a given size, we repeatedly drew 100 random gene sets of a given size. The statistics from these random sets were used to develop the following statistic for assessing the statistical significance.

Given a gene subset of size *n *and its graph-based statistic *y_s_*, we represent distribution of random gene sets of size *n *with a Gaussian distribution defined by two parameters: the means (*μ_n_*) and variances (*σ*_n_^2^). We use the Nadaraya-Watson non-parametric regression [26,28] to capture the non-linear relationship between the size *n *and the parameters of random gene sets of the same size, using the following equations:

(9) $${\hat{\mu }}_{n}=\frac{\sum_{i=1}^{\left|D\right|}w_{n}\left(n_{i}\right)y_{i}}{\sum_{i=1}^{\left|D\right|}w_{n}\left(n_{i}\right)},$$

(10) $${{\hat{\sigma }}^{2}}_{n}=\frac{\sum_{i=1}^{\left|D\right|}w_{n}\left(n_{i}\right){\left(y_{i}-{\hat{\mu }}_{n}\right)}^{2}}{\sum_{i=1}^{\left|D\right|}w_{n}\left(n_{i}\right)},$$

(11) $$w_{n}\left(n_{i}\right)=\exp\left[-\frac{1}{h}{\left(n_{i}-n\right)}^{2}\right],$$

where *D *is the entire dataset of calculated lengths, $\frac{w_{n}\left(n_{i}\right)}{\sum_{i=1}^{\left|D\right|}w_{n}\left(n_{i}\right)}$ is weight for the *i*-th observation, and *w_n _*is the Gaussian kernel function with the bandwidth parameter *h *[26]. The *h *was set to 10 for the regression calculations in this study.

We then assess whether the statistic *y_s _*of a gene set of size *n *belongs to the population of random gene sets defined by *μ_n _*and *σ*_n_^2^, and calculated a *p*-value to reflect the significance. The *p*-value for a new gene set with statistic *y_n _*was calculated as the probability that *y_n _*belongs to the Gaussian distribution defined by parameters *μ_n _*and *σ*_n_^2^, which can be calculated using the distribution function as follows:

(12) $$F\left(y_{n}\right)=\frac{1}{\sqrt{2\pi }}\overset{y_{n}}{\underset{-\infty }{\int }}e^{-{\left(y_{n}-\mu_{n}\right)}^{2}/2\sigma_{n}^{2}}.$$

These calculations were carried out using the Python package rpy2 http://rpy.sourceforge.net/, which uses the statistical language R http://www.r-project.org/.

### Assessing discriminative power of statistical models

To determine which combination of the aforementioned information loss metrics and statistical schemes is the best in terms of differentiating a coherent gene set from a random gene set, we performed a series of experiments using the human pathways listed in the KEGG database as coherent gene sets; for each pathway, an equal number of genes was randomly selected to use as a non-coherent gene set for comparison. The information loss resulting from merging members of a KEGG pathway or a random gene set was calculated using each metric and statistic combination, and the *p*-value of a gene set that belongs to the population of random gene sets of the same size was also calculated. By setting a cutoff *p*-value, we could classify a gene set as coherent or non-coherent, allowing us to perform ROC analysis and assess the discriminative power of a specific combination of information loss metric and graph-based statistical scheme.

### Protein-protein interaction evaluation

We quantified the ratio of the actual protein-protein interactions (PPI) within a gene set over the maximal possible interactions as another measure reflecting the relatedness of genes within a gene set, with the expectation that a more coherent gene set would have a greater number of PPI. The PPI data was obtained from BioGrid [29], where we used the human data of version 3.1.85. The ratio is calculated using the following equation:

(13) $$R_{PPI}=\frac{I}{g\left(1-g\right)},$$

where *I *is the number of existing interactions in the gene set, and *g *is the number of genes in the gene set.

## Results

### Characterization of different information loss measures

**Figure 2:**
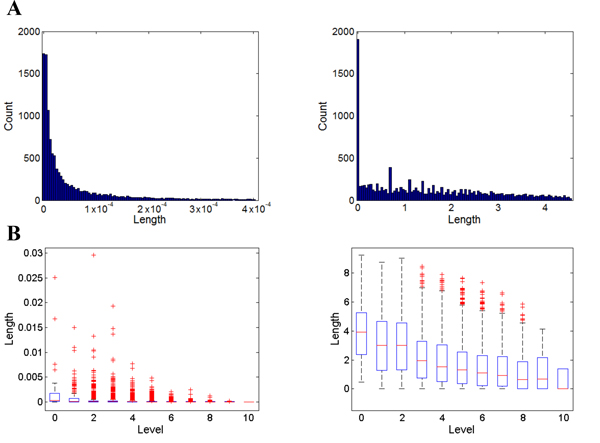
**Distribution of Edge weights**. Both A and B are organized with the IB-based edge weight plot on the left and the IC-based edge weight plot on the right. **A.** Distribution of the shortest 90% of edges in the entire graph. **B.** Boxplots of the edge weight distribution organized according to the level of edge, where level 0 contains edges that connect to the root.

We first set out to assess which of the two information-loss measures, the IC-based and the IB-based, best fit our goal of assessing information loss when genes annotated by highly specific GO terms were merged under a general GO term. We compared the distribution patterns of edge weights represented in different metrics to study their characteristics. Figure 2A shows the histograms of the edges conditioned on edge lengths when calculated using either IB-based (left panel) or IC-based information loss (right panel). The figure shows that the numeric scales of the two metrics are of different orders of magnitude. It also shows that the distributions are mainly dominated by edges with relatively short distances. This was the finding we anticipated, because there are more edges close to the leaf level in the GO hierarchy, where the differences in terms of semantic context or protein information are expected to be small. The distribution of the IB-based edge weights exhibits a smoother transition, whereas the IC-based edge weights demonstrate a peak at edge length zero and a quick drop afterwards, and the distribution contains certain spikes.

In Figure 2B, we plot the distribution of the edge length conditioned on the level of the edge away from the root node. It shows us that the edge length decreases as the node level increases for both IB and IC, indicating the diminishing differences between a pair of parent-child terms when the concepts becomes more specific. The figures also show that the outliers dominate the distributions of the IB-based edge weights at multiple levels; in contrast, the distributions of IC-based edge weights reveal far fewer outliers. Thus, it is apparent that the distribution of IB-based edge weights has a much lower signal-to-noise ratio in comparison to that of IC-based edge weights.

### Evaluation of discriminative powers of graph-based statistics

Since different information-loss measures may perform differently when combined with different statistical schemes, we tried to determine which combination of information-loss measure and graph-based statistical scheme had the highest discriminative power. To this end, we tested whether our model was capable of accurately differentiating between known, functionally coherent gene sets and random gene sets. Using the human KEGG pathways as "functionally coherent" gene sets, we randomly drew for each a matching gene set of the same size as a non-coherent one. For each KEGG pathway of size *n *with a graph-based statistic *y_n_*, we calculated the *p*-value to which the gene set belongs to the population of random gene sets with size *n*. With a *p-*value as the classification threshold, the model classified a gene set as coherent or non-coherent. The discriminative power of the model was then assessed using the area of an ROC curve, reflecting how accurately our models distinguish KEGG pathways from the random gene sets.

**Figure 3:**
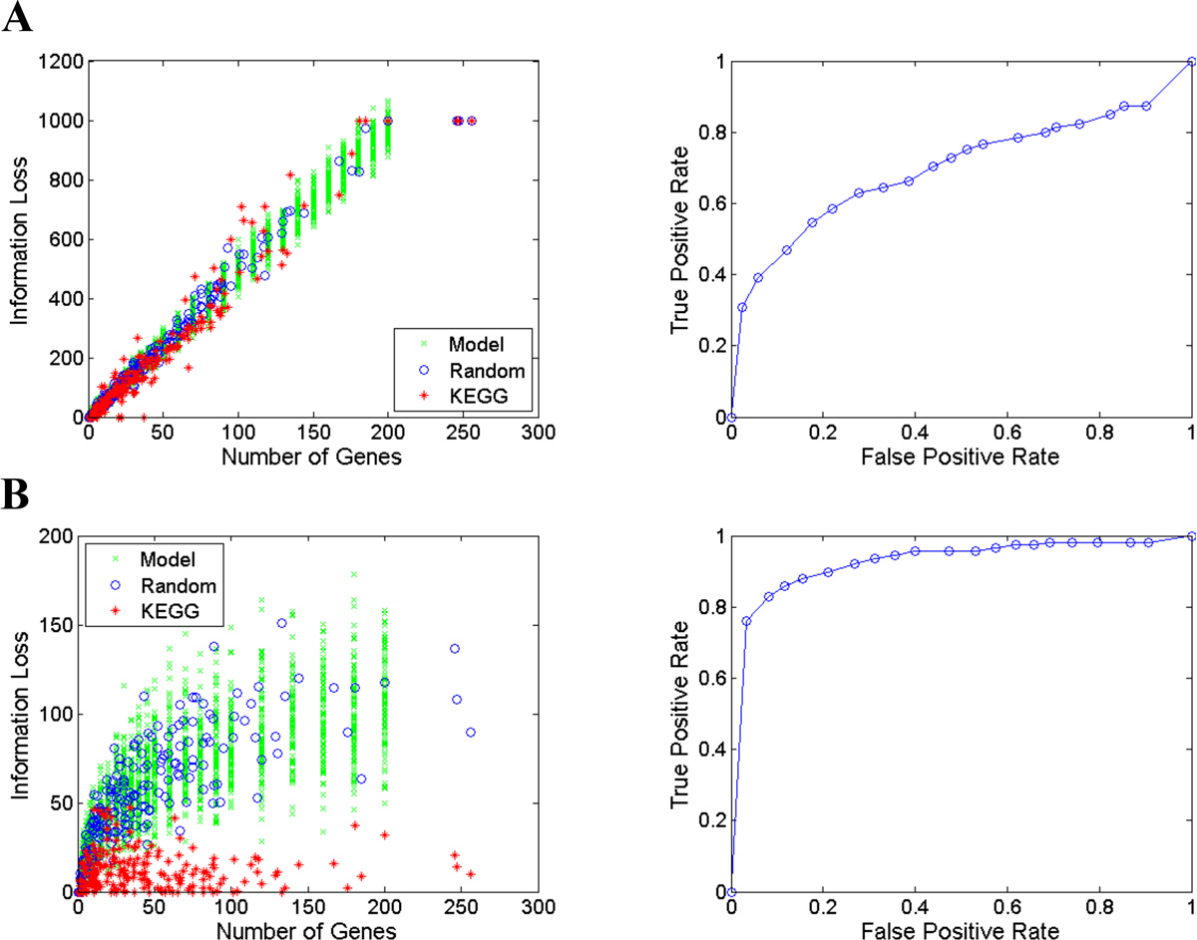
**Example of distributions of graph-based statistics and the discriminative power of coherence models**. **A**. In the scatter plot (top left), statistics derived from KEGG gene sets (red), the matching random gene sets (blue), and simulated random gene sets during model-building (green) were plotted. Top right is the corresponding ROC curve. **B**. Scatter plot of the graph-based statistics and ROC curve of the model using IC as information loss and Steiner tree length derived from augmented GO graph.

Figure 3 shows the results of the analyses of combining IC-based information-loss measures with different graph-based statistical schemes. In Panel A (top two plots), we combined the use of IC as information loss with the length of Steiner trees derived from a GOGeneGraph graph as statistic. The figure shows that this combination cannot separate KEGG pathway gene sets from the matched random and simulated random gene sets. Thus, the discriminative power of the model is poor, as shown in the ROC curve. In contrast, the combination of the use of IC as the information loss metric and the length of Steiner trees derived from *an augmented GOGeneGraph *as statistic exhibited significant differences in the distribution of the data points from the KEGG gene sets and random gene sets. Thus, the corresponding model revealed a much higher discriminative power. The results of ROC analysis of all pairwise combinations of information loss metrics and graph-based statistics schemes are shown in Table 1; they indicate that the combination of IC and Steiner tree from an augmented GO graph performs the best.

**Table 1:** ROC analysis results for information loss metric and statistical scheme combinations.

	Unaugmented Steiner Tree	Augmented Steiner Tree
**Information Bottleneck**	0.7133	0.8363

**Information Content**	0.6983	0.9251

Area under the ROC Curve values for the statistical models based on each combination of information loss metric and statistical scheme.

### Finding multiple function aspects of KEGG pathways

**Figure 4:**
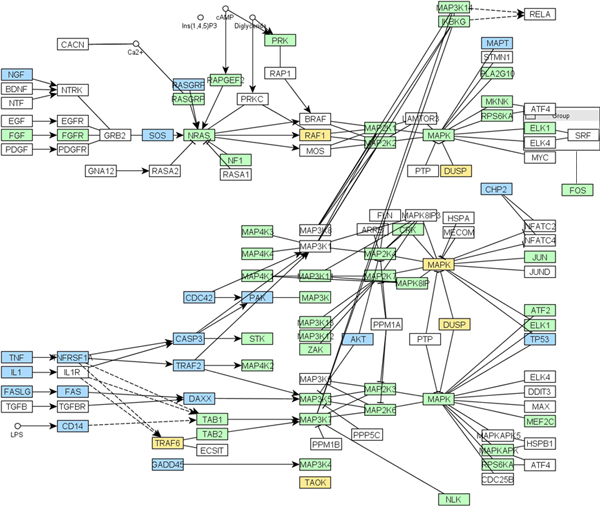
**An example of a KEGG pathway containing genes involved in multiple processes**. The "MAPK signaling pathway" (hsa04010) is shown. Two functionally coherent subsets are highlighted. The genes summarized by GO:0023014 (*Signal transduction by phosphorylation*) are in green; the genes summarized by GO:0006915 (*Apoptotic process*) are in blue. Genes involved in both biological processes are in yellow.

In the previous section, we used the gene sets from the KEGG pathway database as the surrogates of coherent gene sets to compare the discriminative power of statistical models utilizing different combinations of information loss metrics and graph-based statistics. However, we noted that many KEGG pathways contain a large number of genes performing diverse functions, and our model classified them as non-coherent gene sets. Instead of simply treating such calls by our model as errors, we further investigated whether it made sense to treat a large KEGG pathway gene set as a coherence gene set, and whether it is more sensible to use our approach to identify fine-grained, coherent gene sets from such a pathway. Figure 4 shows an example of one such KEGG pathway (*hsa04010*): the human *MAPK signaling pathway*, which includes 262 unique genes (not all are shown in the figure). This KEGG pathway comprehensively includes many cellular signal transduction components sharing the proteins involved in the MAPK cascade, including growth factor signaling pathways and the signaling pathway that induces apoptosis. As such, it may not be biologically sensible or even possible to find an informative concept to summarize the diverse biological processes of genes in this KEGG database entry. Indeed, we tried to search for a GO term to cover all the genes listed in this pathway, a process which led to the most uninformative term of the Biological Processes domain, the root GO term. Therefore, it is sensible that our model treated the whole set of genes as not coherently related. When applying our method to the gene list of this pathway to search for coherent subsets, our model returned a number of non-disjoint gene subsets, reflecting different aspects of the functions in which these genes participate. For example, one subset was summarized by the GO term GO:0023014 (*signal transduction by phosphorylation*), which included most of the genes in the pathway that are involved in the protein phosphorylation process, including MAPK kinases, and are shown as the genes in green. Another facet of the functions of these genes was summarized by the GO term GO:0006915 (*apoptosis*), which included many genes with well-known roles in the process of apoptosis, and are shown as the genes in blue. Thus, in this figure, these two concepts reflect two functional themes of the genes with a suitable level of specificity and generality; the rest of the concepts representing different functional themes are listed in Additional File 1.

### Application in real world data analysis

We then investigated the application of our method in a real-world data analysis experiment to illustrate the challenges a bioinformatician often faces when using conventional functional analysis to detect functional themes from a long gene list, and to show the advantages of our approaches.

Cancers result from genomic perturbations that lead to changed cellular signal pathways. A common manifestation of a perturbation in a signaling pathway is the altered expression of modules of genes that carry out specific biological processes [30]. Another important characteristic of cancers is that a tumor results from a combination of perturbations in multiple signaling pathways, thus manifesting as the perturbed expression of genes involved in multiple biological processes [2]. As such, a list of differentially expressed genes from a cancer sample represents a mixture of responses to different signal perturbations; therefore, de-convoluting the biological processes reflected in such a list of differentially expressed genes is a critical task.

**Figure 5:**
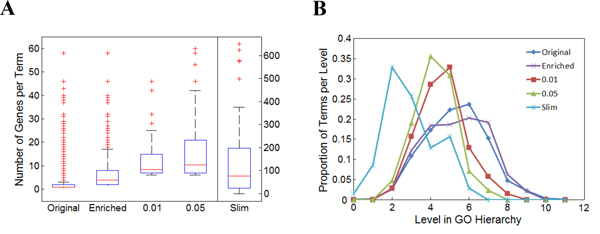
**Distribution of genes associated with summarizing GO terms**. **A**. Boxplots of the distribution of number of genes associated per term under five different conditions: original GO annotation; enriched GO annotations; our method with a p-value ≤ 0.01 and 0.05 thresholds; and the Generic GO slim. **B**. Plot of the proportion of GO terms per level in the GO hierarchy under the five different conditions.

As a concrete example, we identified a list of genes from the ovarian cancer samples collected from TCGA. The list included a total of 837 genes, each of which was deferentially expressed in at least 5 tumor samples [31]. We first set out to evaluate whether it was suitable to use the original GO annotations, GO annotation enrichment analysis, and GO slim mapping to identify function themes. We found that a total of 2,175 unique GO terms from the BP domain were associated with the genes in the list, and that the median number of genes annotated by these GO terms was 1. The distribution of the number of genes annotated by these terms is shown as a box plot, labeled as "original", in Figure 5A. We then performed a conventional hypergeometric-distribution-based GO term enrichment analysis to identify the "enriched" GO terms at a cutoff *p-*value of 0.05. The analysis resulted in a set of 433 unique GO terms, the median number of genes annotated by which was 4 (see Figure 5A). Since the size of gene modules associated with each enriched GO term appeared to be too small to represent the "major" themes, we further studied the genes mapped to the human GO slim terms, finding a total of 70 human GO slim terms, the median number of genes mapped to which was 75.

When applying our method to the gene list, we can use different *p*-values as cutoffs for grouping genes into subsets, with a smaller *p-*value associated with a set whose genes are more coherently related to one another. Using a cutoff *p-*value at 0.01, our method identified 70 subsets deemed to be functionally coherent; the median of the number of genes within these subgroups was 8 (see Figure 5A). When we relaxed the information loss requirement by setting the cutoff *p-*values at 0.05, we identified fewer modules as more genes were merged into these subsets; the median number of genes covered by these terms was 10.

To investigate the degree of specificity of the summarizing GO terms derived from different methods, we plotted their distribution based on their level, i.e., the number of steps away from a root a GO term was found to be (see Figure 5B). The results show that the GO slim terms tend to be very close to the root; thus, in comparison to other methods, these concepts tend to be very general, and lacking in specific information. The figure also shows that the original and "enriched" terms broadly span the GO hierarchy, with more terms concentrated at very specific levels. Combining the information conveyed by the two panels, one can draw the conclusion that the original and "enriched" GO terms tend to be highly specific, annotating only a small number of genes. In comparison, the summarizing GO terms identified by our methods tend to be more specific than the GO slims but more general than the original and "enriched" annotations, with the terms concentrating at levels 5 and 6, and achieving a better balance between the number of genes covered and the specificity of the terms.

**Figure 6:**
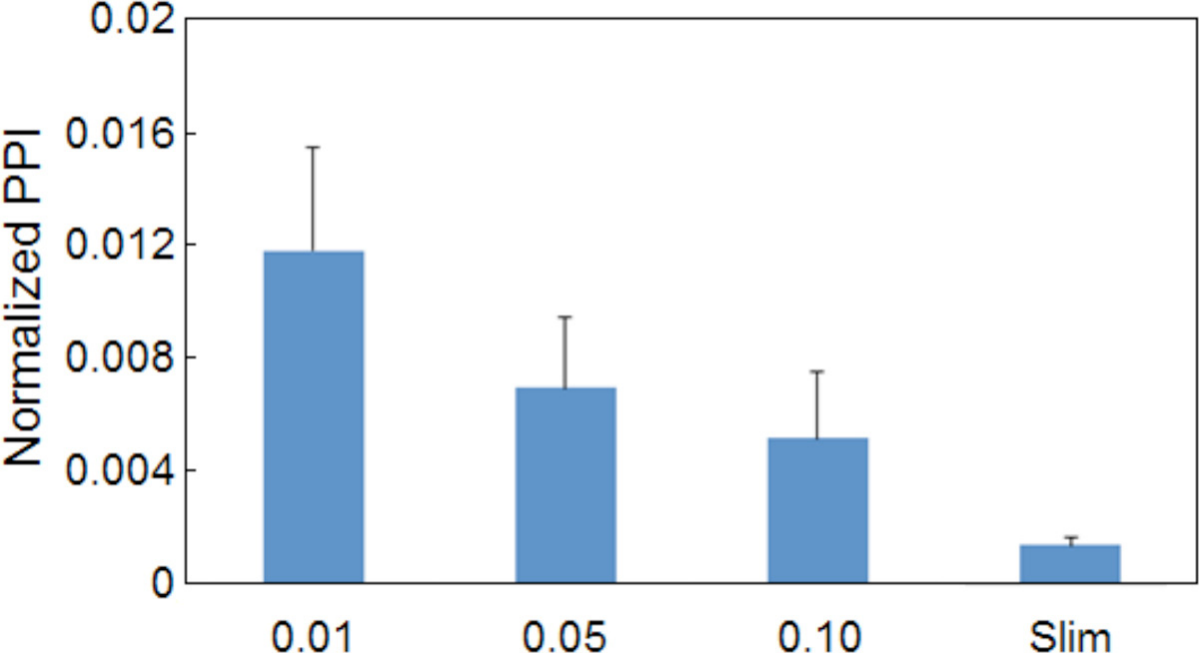
**Average within-module PPI ratios for summarizing terms**. Plots of the calculated average within-module PPI ratio for the summarizing terms that resulted from different thresholds of merging and use of the AMIGO GO Slim tool. The whisker denotes the calculated standard error.

To support the notion that the statistical model developed by this study effectively measures the functional coherence, we assessed the functional relatedness of the proteins in a subset returned by our models using another measure, the within-module PPI ratio, and compared the results. In a series of experiments, we applied our model to the differentially expressed gene set using 3 different *p-*value cutoff thresholds (0.1, 0.05 and 0.01), leading to 3 collections of modules. We then investigated whether the within-module PPI ratio exhibited an anti-correlation with *p-*values, based on the assumption that a more coherent gene set (with a smaller *p-*value) should generate more within-module PPIs. Figure 6 plots the within-module PPI ratios of the gene modules derived using different *p-*value cutoff thresholds, as well as the value derived from the modules produced by mapping genes to the Generic GO slim. The results indicate that, indeed, the more stringent the *p-*values, the higher the within-module PPI ratio; the genes grouped by the GO slim had the lowest within-module PPI ratio. Our metric agrees with another biologically sensible metric reflecting the functional coherence of genes. A similar finding by Dutkowski et al. [32] corroborates our results as well.

## Discussion and conclusion

The goal of this study was to develop and evaluate approaches that can be used to conceptualize molecular findings and capture the major functional themes of genes. Conceptualization of information from experimental data is a critical step toward deriving knowledge from data [33]. We assessed different metrics to measure information loss and its combination with different schemes to derive statistics reflecting the overall information loss that would result from grouping genes under a summarizing concept in the Gene Ontology. Our results indicate that the best discriminative power was achieved by combining the IC-based information-loss metric with the statistics derived from the Steiner tree connecting the genes on an augmented GO graph. We further demonstrate that our methods provide a novel approach to revealing major functional themes of a list of genes in an objective and quantitative fashion.

It is worth pointing out that, while the IC-based metric has been widely used to reflect the "semantic" similarity of GO terms [22,23,34,35], the metric does not really reflect the *semantic *content, but rather represents the amount of information a GO term reveals with respect to genes. As such, the difference of ICs between two terms reflects the difference in the information with respect to genes; therefore, the phrase "information loss with respect to genes" would be more appropriate terminology for the quantity. In previous work, we proposed to use the word-usage profile of the biomedical literatures in which the concept is encoded by a GO term to represent the semantic context of a GO concept; we further proposed the use of the IB-based metric to measure the difference in the semantic context of GO terms. Since this metric is based on natural language words, the tokens conveying semantic meanings for humans, we reasoned it should be a more suitable metric for assessing how closely the functions of genes are related to each other based on the annotation. The smooth distribution of edge weights measured with the IB-based metric seemingly supported the notion that the metric measures a gradual change of semantic difference between GO terms. However, one of the premises for this metric to be stable is that each GO node is associated with a large number of documents in order to produce a representative word-usage profile--a premise which was not borne out for many GO terms in our case. Instead, we observed a relatively small signal-to-noise ratio for the metric, rendering it inferior in terms of discriminating known coherent gene sets from random ones. A possible approach is to either collect more training documents associated with GO terms or to design new metrics based on a word-usage profile that is relatively stable. Nonetheless, since the ultimate goal of the IC-based and IB-based metrics is to provide discriminative power for differentiating coherent gene sets from non-coherent ones, our experiments indicate that the IC-based metric is up to the task, and that all information contained by a GO term is of value.

## Competing interests

The authors declare that they have no competing interests.

## Authors' contributions

XL conceived of the study, and both XL and VC participated in its design. VC performed the experiments. XL and VC drafted the manuscript. Both authors read and approved of the final manuscript.

## Supplementary Material

Additional file 1 **Gene Ontology terms summarizing the MAPK signaling pathway**. Click here for file

## Declarations

The publication cost is covered with the grant R01 LM 011155.

## References

[B1] TCGA Research Network Integrated genomic analyses of ovarian carcinoma Nature 2011 7 7353 609 615 10.1038/nature10166 21720365 PMC3163504

[B2] Hanahan D Weinberg RA Hallmarks of cancer: the next generation Cell 2011 7 5 646 674 10.1016/j.cell.2011.02.013 21376230

[B3] Arnaud MB Costanzo MC Shah P Skrzypek MS Sherlock G Gene Ontology and the annotation of pathogen genes: the case of *Candida albicans* Trends in Microbiology 2009 7 7 295 303 10.1016/j.tim.2009.04.007 19577928 PMC3907193

[B4] Thomas PD Mi H Lewis S Ontology annotation: mapping genomic regions to biological function Current Opinion in Chemical Biology 2007 7 1 4 11 10.1016/j.cbpa.2006.11.039 17208035

[B5] McCarthy FM Mahony TJ Parcells MS Burgess SC Understanding animal viruses using the Gene Ontology Trends in Microbiology 2009 7 7 328 335 10.1016/j.tim.2009.04.006 19577474

[B6] Giglio MG Collmer CW Lomax J Ireland A Applying the Gene Ontology in microbial annotation Trends in Microbiology 2009 7 7 262 268 10.1016/j.tim.2009.04.003 19577473

[B7] Chibucos MC Tseng TT Setubal JC Describing commonalities in microbial effector delivery using the Gene Ontology Trends in Microbiology 2009 7 7 312 319 10.1016/j.tim.2009.05.001 19576779

[B8] Ashburner M Ball C Blake J Botstein D Butler H Cherry M Davis A Dolinski K Dwight S Eppig J Gene Ontology: tool for the unification of biology Nature Genetics 2000 7 1 25 29 10.1038/75556 10802651 PMC3037419

[B9] Huang DW Sherman BT Lempicki RA Bioinformatics enrichment tools: paths toward the comprehensive functional analysis of large gene lists Nucleic Acids Research 2009 7 1 1 13 10.1093/nar/gkn923 19033363 PMC2615629

[B10] Huang DW Sherman BT Lempicki RA Systematic and integrative analysis of large gene lists using DAVID Bioinformatics Resources Nature Protocols 2009 7 1 44 57 19131956 10.1038/nprot.2008.211

[B11] Zhang B Kirov S Snoddy J WebGestalt: an integrated system for exploring gene sets in various biological contexts Nucleic Acids Research 2005 7 Web Server W741 W748 10.1093/nar/gki475 15980575 PMC1160236

[B12] Khatri P Draghici S Ostermeier GC Krawetz SA Profiling gene expression using Onto-express Genomics 2002 7 2 266 270 10.1006/geno.2002.6698 11829497

[B13] Hu ZL Bao J Reecy JM CateGOrizer: A Web-Based Program to Batch Analyze Gene Ontology Classification Categories Online Journal of Bioinformatics 2008 7 2 108 112

[B14] McCarthy FM Wang N Magee GB Nanduri B Lawrence ML Camon EB Barrell DG Hill DP Dolan ME Williams WP AgBase: a functional genomics resource for agriculture BMC Genomics 2006 7 229 10.1186/1471-2164-7-229 PMC1618847 16961921

[B15] Carbon S Ireland A Mungall CJ Shu S Marshall B Lewis S Hub A Group WPW AmiGO: online access to ontology and annotation data Bioinformatics 2009 7 2 288 289 10.1093/bioinformatics/btn615 19033274 PMC2639003

[B16] Kawashima S Katayama T Sato Y Kaneshia M KEGG API: a web service using SOAP/WSDL to access the KEGG system Genome Informatics 2003 7 673 674

[B17] Subramanian A Tamayo P Mootha VK Mukherjee S Ebert BL Gillette MA Paulovich A Pomeroy SL Golub TR Lander ES Gene set enrichment analysis: A knowledge-based approach for interpreting genome-wide expression profiles Proceedings of the National Academy of Sciences of the United States of America 2005 7 43 15545 15550 10.1073/pnas.0506580102 16199517 PMC1239896

[B18] Muller B Richards AJ Jin B Lu X GOGrapher: A Python library for GO graph representation and analysis BMC research notes 2009 7 122 10.1186/1756-0500-2-122 19583843 PMC2714316

[B19] Jin B Lu X Identifying informative subsets of the Gene Ontology with information bottleneck methods Bioinformatics 2010 7 18 2445 2451 20702400 10.1093/bioinformatics/btq449 PMC2944202

[B20] Tishby N Pereira FC Bialek W The Information Bottleneck Method Proceedings of the 37th Annual Allerton Conference on Communication, Control and Computing 368 377

[B21] Slonim N Tishby N Document Clustering using Word Clusters via the Information Bottleneck Method Proceedings of the 23rd Annual International ACM SIGIR Conference on Research and Development in Information Retrieval 2000 208 215

[B22] Lord PW Stevens RD Brass A Goble CA Investigating semantic similarity measures across the gene ontology: The relationship between sequence and annotation Bioinformatics 2003 7 10 1275 1283 10.1093/bioinformatics/btg153 12835272

[B23] Resnik P Using Information Content to Evaluate Semantic Similarity in a Taxonomy Proceedings of the 14th International Joint Conference on Artificial Intelligence 1995 448 453

[B24] Lin D An information-theoretic definition of similarity Proceedings of the 15th International Conference on Machine Learning 1998 296 304

[B25] Jiang JJ Conrath DW Semantic similarity based on corpus statistics and lexical taxonomy Proceedings of the 10th International Conference on Research on Computational Linguistics 1997

[B26] Richards AJ Muller B Shotwell M Cowart LA Rohrer B Lu X Assessing the functional coherence of gene sets with metrics based on the Gene Ontology graph Bioinformatics 2010 7 12 i79 i87 10.1093/bioinformatics/btq203 20529941 PMC2881388

[B27] Kou LT Markowsky G Berman L A fast algorithm for Steiner trees Acta Informatica 1981 7 2 141 145 10.1007/BF00288961

[B28] Nadarya EA On estimating regression Theory of Probability and its Applications 1964 7 1 141 142 10.1137/1109020

[B29] Stark C Breikruetz B-J Reguly T Boucher L Brietkreutz A Tyers M BioGRID: a general repository for interaction datasets Nucleic Acids Research 2006 7 Database Issue D535 539 16381927 10.1093/nar/gkj109 PMC1347471

[B30] Segal E Friedman N Koller D Regev A A module map showing conditional activity of expression modules in cancer Nature Genetics 2004 7 10 1090 1098 10.1038/ng1434 15448693 PMC2271138

[B31] Lu S Lu X Integrating genome and functional genomics data to reveal perturbed signaling pathways in ovarian cancers AMIA Summits Translational Science Proceedings 2012 2153-4063 (Electronic) 72 78 PMC3392049 22779056

[B32] Dutkowski J Kramer M Surma M Balakrishnan R Cherry M Krogan N Ideker T A gene ontology inferred from molecular networks Nature Biotechnology 2012 7 1 38 45 10.1038/nbt.2463 PMC3654867 23242164

[B33] Lu S Jin B Cowart LA Lu X From data towards knowledge: Revealing the architecture of signaling systems by unifying knowledge mining and data mining of systematic perturbation data PLoS ONE 2013 10.1371/journal.pone.0061134 PMC3634064 23637789

[B34] Sevilla JL Segura V Podhorski A Guruceaga E Mato JM Martinez-Cruz LA Corrales FJ Angel R Correlation between Gene Expression and GO Semantic Similarity IEEE/ACM Transactions on Computational Biology and Bioinformatics 2005 7 4 330 338 10.1109/TCBB.2005.50 17044170

[B35] Pesquita C Faria D Falcão AO Lord P Couto FM Semantic Similarity in Biomedical Ontologies PLoS Computational Biology 2009 7 7 1 12 10.1371/journal.pcbi.1000443 PMC2712090 19649320

