# Supplementary material for: Conceptualization of molecular findings by mining gene annotations
Source: BMC Proc. 2013 Dec 20;7(Suppl 7):S2. doi: 10.1186/1753-6561-7-S7-S2 (PMC4042834; doi:10.1186/1753-6561-7-S7-S2)
Supplement: Additional file 1 — Gene Ontology terms summarizing the MAPK signaling pathway. [file 1753-6561-7-S7-S2-S1.docx]

## Gene Ontology terms summarizing the MAPK signaling pathway

| **GO ID** | ***P*-values** | **Gene Count** | **Term Name** |
| --- | --- | --- | --- |
| GO:0007166 | 7.10E-06 | 108 | cell surface receptor linked signaling pathway |
| GO:0006796 | 1.90E-05 | 75 | phosphate metabolic process |
| GO:0023014 | 2.73E-05 | 75 | signal transduction via phosphorylation event |
| GO:0007596 | 1.69E-04 | 36 | blood coagulation |
| GO:0007411 | 0.000222 | 37 | axon guidance |
| GO:0010941 | 0.000369 | 49 | regulation of cell death |
| GO:0051338 | 0.00037 | 71 | regulation of transferase activity |
| GO:0045087 | 0.000402 | 41 | innate immune response |
| GO:0002758 | 0.00041 | 40 | innate immune response-activating signal transduction |
| GO:0090047 | 0.000489 | 16 | positive regulation of transcription regulator activity |
| GO:0042127 | 0.000495 | 39 | regulation of cell proliferation |
| GO:0006915 | 0.000508 | 28 | apoptosis |
| GO:0009893 | 0.000535 | 22 | positive regulation of metabolic process |
| GO:0042221 | 0.00058 | 15 | response to chemical stimulus |
| GO:0030168 | 0.00062 | 23 | platelet activation |
| GO:0007268 | 0.000655 | 26 | synaptic transmission |
| GO:0060255 | 0.000822 | 14 | regulation of macromolecule metabolic process |
| GO:0051270 | 0.00092 | 16 | regulation of cellular component movement |
| GO:0090304 | 0.000923 | 36 | nucleic acid metabolic process |
| GO:0019219 | 0.000948 | 45 | regulation of nucleobase, nucleoside, nucleotide and nucleic acid metabolic process |
| GO:0034097 | 0.001301 | 14 | response to cytokine stimulus |
| GO:0006954 | 0.00141 | 13 | inflammatory response |
| GO:0009966 | 0.001554 | 57 | regulation of signal transduction |
| GO:0035556 | 0.002017 | 50 | intracellular signal transduction |
| GO:0006112 | 0.002284 | 12 | energy reserve metabolic process |
| GO:0009887 | 0.002284 | 12 | organ morphogenesis |
| GO:0051726 | 0.00232 | 15 | regulation of cell cycle |
| GO:0044267 | 0.002405 | 23 | cellular protein metabolic process |
| GO:0006935 | 0.002854 | 11 | chemotaxis |
| GO:0071260 | 0.002854 | 11 | cellular response to mechanical stimulus |
| GO:0002764 | 0.002932 | 12 | immune response-regulating signaling pathway |
| GO:0006753 | 0.002948 | 10 | nucleoside phosphate metabolic process |
| GO:0007399 | 0.002948 | 10 | nervous system development |
| GO:0043434 | 0.003074 | 40 | response to peptide hormone stimulus |
| GO:0031323 | 0.005161 | 26 | regulation of cellular metabolic process |
| GO:0007267 | 0.006999 | 21 | cell-cell signaling |
| GO:0009653 | 0.007015 | 20 | anatomical structure morphogenesis |
| GO:0006950 | 0.007145 | 23 | response to stress |
| GO:0030154 | 0.007304 | 19 | cell differentiation |
| GO:0006812 | 0.010204 | 12 | cation transport |
| GO:0008150 | 0.010395 | 16 | biological_process |
| GO:0080134 | 0.012309 | 27 | regulation of response to stress |
| GO:0048856 | 0.012357 | 15 | anatomical structure development |
| GO:0007165 | 0.021096 | 71 | signal transduction |
| GO:0051336 | 0.023724 | 21 | regulation of hydrolase activity |
| GO:0050789 | 0.026565 | 13 | regulation of biological process |
| GO:0048646 | 0.026565 | 13 | anatomical structure formation involved in morphogenesis |
| GO:0051049 | 3.61E-02 | 47 | regulation of transport |
| GO:0022603 | 0.036737 | 15 | regulation of anatomical structure morphogenesis |
| GO:0051128 | 0.041248 | 12 | regulation of cellular component organization |
| GO:0007010 | 0.047174 | 16 | cytoskeleton organization |
